# Roll out of intraveneous artesunate under named patient programmes in the Netherlands, Belgium and France

**DOI:** 10.1186/1750-1172-8-150

**Published:** 2013-09-24

**Authors:** Annemarie Rosan Kreeftmeijer-Vegter, Cornelis KW van Veldhuizen, Peter J de Vries

**Affiliations:** 1 Department of Internal Medicine, Division of Infectious Diseases, Academic Medical Centre, Meibergdreef 9, 1105, AZ Amsterdam, The Netherlands; 2 ACE Pharmaceuticals BV, Schepenveld 41, 3891, ZK Zeewolde, The Netherlands; 3 Department of Internal Medicine, Tergooi Ziekenhuizen, Van Riebeeckweg 212, 1213, XZ Hilversum, The Netherlands

**Keywords:** Named patient programme, Legislation, Roll out, Pharmacovigilance, IV artesunate

## Abstract

**Background:**

Intravenous (IV) artesunate is the treatment of choice for severe malaria. In Europe, this treatment is only available in a few countries via named patient programmes (NPPs). As a case study, the legal and organisational aspects and pharmacovigilance of these NPPs and possibilities for harmonisation within the EU were studied over time and space using IV artesunate (Malacef) in the Netherlands, Belgium and France.

**Methods:**

The legal base and organisation of NPPs in the Netherlands, Belgium and France were studied. The diffusion and cumulative availability of IV artesunate and the pharmacovigilance components were compared among the three countries using distribution data from the period 2007 through 2012.

**Results:**

Artesunate has quickly gained acceptance for treating severe malaria in the Netherlands, whereas both Belgium and France have introduced this treatment more hesitantly. This difference in acceptance is due to differences in the implementation of NPP legislation among the countries. France currently has a proactive system in which treatment requires the permission for each patient and an intensive follow-up protocol. On the other hand, Belgium and Dutch NPPs are more dependent on the investigators’ initiative and are therefore potentially faster and more flexible, facilitating the discovery of adverse effects that have not been reported by more formal comparative clinical trials.

**Conclusions:**

NPPs provide a unique opportunity to study both the benefits and risks of unregistered products for treating rare diseases, provided that the patients are actively vigilated. Thus, we recommend that NPPs should be harmonised throughout Europe in order to ensure equal availability of treatment and therapeutic benefit to all Europeans without compromising patient safety.

## Background

Severe malaria is a medical emergency that has a mortality rate of nearly 100% if not treated promptly [1]. Artesunate is the current drug of choice for treating severe malaria [1]. The anti-malarial properties of artesunate are superior to quinine, and artesunate significantly reduced mortality among both children and adults in the two largest trials conducted to date for treating severe falciparum malaria [2,3].

Despite its clear advantages over other treatments, IV artesunate is not currently available in either Europe or the United States, and this is due to two separate issues. Firstly, whether evidence obtained from malaria-endemic regions can be generalised to the European population is a subject of debate. Moreover, whether it is justified, feasible and necessary to conduct comparative studies for European patients given the currently available evidence and the recommendation by the World Health Organization (WHO) to use artesunate as the drug of choice for treating severe malaria [1,4] is also under discussion. Secondly, artesunate is not manufactured in accordance with European Good Manufacturing Practice (EU-GMP), which is a requirement for market authorisation. The WHO recently prequalified the Chinese version of this product [5] after the manufacturer (Guilin Pharmaceuticals Company, Shanghai, China) improved their production process, thus ensuring that the drug is manufactured in compliance with WHO-GMP requirements. Nevertheless, this standard is not considered to reach the same GMP level as the EU-GMP. In both the EU and the United States, IV artesunate does not have market authorisation yet.

Although marketing and supplying unauthorised pharmaceutical products are prohibited in the EU, access to drugs prior to their formal approval may be granted for individual patient care, provided no currently registered alternative is available within the EU. The European legal framework provides two situations in which non-licenced medical products can be given to patients; these situations are compassionate-use programmes (CUPs) and named patient programmes (NPPs) [6].

CUPs apply to a group of patients with a medical condition for which a drug therapy is being studied and who do not (or no longer) participate in the study and who wish to be treated with the investigational drug. NPPs, as the name suggests, refer to the use of a drug in an individual patient and are the responsibility of the prescribing physician. Although a regulatory framework exists for access to unauthorised medicinal products at the European level, the approval, implementation and oversight of such programmes remain the responsibility of the individual country.

Prior to 2007, IV artesunate was not available in Europe, despite an increasing demand from physicians who were treating malaria. In response to this demand, clinicians at the Academic Medical Centre, Amsterdam, the Netherlands (including Dr. P.J. de Vries) initiated an NPP in collaboration with ACE Pharmaceuticals BV (in Zeewolde, the Netherlands), a company that specialises in orphan drugs and medical-need products, after permission was granted by the Dutch Health Care Inspectorate. IV artesunate is imported from Guilin (Shanghai, China) by ACE Pharmaceuticals; following an extensive series of quality and sterility controls, the drug is then released for distribution under the trade name Malacef® 60. The product is kept as an emergency stock in the hospital pharmacies and is prescribed for individual patients and accompanied by a medical statement from the prescribing physician. In parallel with this NPP, in February 2007 an orphan designation (EU/3/07/430) was granted by the European Commission to ACE Pharmaceuticals for using IV artesunate to treat malaria.

The objective of this study was to describe and compare the availability of IV artesunate over time and space under named patient programmes in the Netherlands and two additional European countries (Belgium and France) from 2007 through 2012, with the overall aim of identifying patterns that will be useful for harmonising NPPs throughout the EU.

## Methods

### Legal basis and description of implementation of NPPs in the Netherlands, Belgium and France

In Europe, the legal framework for NPPs i.e. the supply of unlicensed medicines for use by individual patients is governed by EU member countries as described in Article 5 of Directive 2001/83/EC. The implementation and regulatory requirements of such programmes vary widely among EU countries. The national legislation that governs NPPs and their implementation in the Netherlands, Belgium and France, the three countries in which Malacef is available through an NPP, was studied by searching for Internet-based documents on national government websites and websites of national authorities or the agencies responsible for decision-making with respect to medicines and healthcare products. The search was performed in January 2013.

### Availability of Malacef in the Netherlands, Belgium and France

The process of making Malacef available to prescribers and the pharmacovigilance of Malacef were studied by requesting data from the supplier and from pharmaceutical and medical professionals. ACE Pharmaceuticals provided pharmaceutical distribution data regarding Malacef in the Netherlands, Belgium and France. The presence of artesunate treatment for at least one adult patient with severe malaria was used as a measure of availability. Cumulative availability was calculated using distribution data and was grouped according to the following three hospital types: university medical centres (UMCs), teaching hospitals (i.e. general hospitals affiliated with a university medical centre) and general hospitals. In France, because no distinction exists between the first two hospital types, French UMCs and teaching hospitals were grouped under the heading Centre Hospitalier Universitaire (CHU). To allow a direct comparison between countries, the availability of Malacef in the Netherlands, Belgium and France was expressed per population capita by dividing the number of Malacef vials ordered by each country by that country’s total population; for plotting the data, the resulting value was then multiplied by 1000.

### Pharmacovigilance

Pharmacovigilance data were proactively collected as previously described [7] for all Dutch patients who were treated with IV artesunate in the period from November 2007 through December 2010 and for all Belgian patients who were treated in the period from January 2009 through December 2010. For French patients, safety data were received from the *Agence National de Securité du Medicament et des Produits de Santé* (ANSM), the national French authority in charge of, among others, pharmacovigilance.

## Results

### Legal basis and implementation of NPPs in the Netherlands, Belgium and France

For the Netherlands, the legal basis of NPPs is laid down in Article 3.17 of the Regulation of the Dutch Medicines Act [8]. The competent Dutch authority that is responsible for decision-making regarding medicines and healthcare products is the CBG-MEB (Medicines Evaluation Board) [9]. Because dispensing a medicinal product on the basis of a physician’s statement (stating a named patient, the indication and the product name) falls under the jurisdiction of the Health Care Inspectorate, we also accessed their website [10].

In Belgium, an NPP can be implemented in accordance with the law regarding medicinal products of 25/03/1964 [11], revised on 1 May, 2006 (art.6 quarter §1, 1°), and its Royal Decree dated 14/12/2006 (art. 102 and 105). The Federal Agency for Medicines and Health Products (FAMHP) [12] is the competent Belgian authority responsible for the quality, safety and efficacy of medicines and healthcare products*.*

In France, using medicinal products that lack marketing authorisation is conditioned upon first obtaining a Temporary Authorisation for Use (ATU) from the ANSM, formerly known as AFSSAPS [13]. The general principles guiding the ATU and its legal provision were already laid down in 1994.

The additional requirements for the implementation and pharmacovigilance of an NPP in all three countries are summarised in Table 1.

**Table 1:** Implementation of NPPs in the Netherlands, Belgium and France

**Category**	**Determinants**	**The Netherlands**	**Belgium**	**France**
Roll out	National legislation	Article 3.17 of the Dutch Regulation concerning Medicines Act (Regeling Geneesmiddelenwet)	Article 6 quarter §1, 1° of the law on medicinal products of 25/03/1964, revised on 01.05.2006 and its Royal Decree dated 14/12/2006 (Art. 102 and 105).	Article L.5121-12 a of the French Public Health Code
	Jurisdiction	Health Care Inspectorate (IGZ)	FAMHP (Federal Agency for Medicines and Health Products)	ANSM (French National Agency for Medicines and Health Products Safety; under the supervision of the Ministry of Health).
	Legal entities that can apply for an NPP	-Manufacturers	The licence-holder as defined in Art 74 of the Royal Decree dated 14/12/2006.	Request of the prescribing physician submitted to ANSM by a pharmacist at the healthcare institution.
		-Wholesalers		
		-Pharmacists		
		-Dispensing general practitioners		
		The applicant must be domiciled or have a registered office in the Netherlands.		
	Application	Application must be accompanied by documents to enable assessment of safety, therapeutic rationale and (non-) substitutability of the product. The first application must be accompanied by a declaration signed by the prescribing medical doctor in which the doctor agrees to be fully responsible and to accept the risks of prescribing an unlicensed medicinal product.	Authorisation for importation, distribution and delivery of unauthorised medicinal products is obtained through an unsolicited request (medical statement) from a healthcare professional for use in a particular patient. The initiation and conduct of the treatment falls under the full responsibility of the treating physician.	A ‘nominative’ ATU is issued for a named patient, and upon the request and responsibility of the prescribing physician. The request form is accompanied by a prescription, the patient’s clinical history and a justification for using the drug, and is submitted by the hospital pharmacist. ANSM must authorise the ATU for each patient and reserves the right to modify or discontinue the ATU at any time.
	Entity that can supply the drug	Manufacturers , wholesalers, pharmacists, dispensing general practitioners	The licence-holder as defined in Art 74 of the Royal Decree dated 14/12/2006 at the request of a pharmacist based on a prescription for a properly defined patient.	Only hospital pharmacists may supply the medicinal product subject to an ATU.
Pharmacovigilance	Entity demanding pharmacovigilance	The Inspectorate	The Belgian Centre for Pharmacovigilance for medicines for Human use (BCPH), which is part of the FAMHP.	ANSM
	Patient follow-up and data collection	The Inspectorate asks suppliers to report annually the number of patients treated with the specified unregistered product	Unspecified	ANSM requires a medicinal product dossier beforehand from the pharmaceutical company in order to establish a predefined protocol for therapeutic use (PTU), specifying the conditions for use, patient follow-up and the collection of efficacy and safety data.
	Reporting adverse events	Adverse reactions suspected to be related to the medicinal product must be reported to the Inspectorate by the treating physician (specified in the doctor’s declaration)	Adverse reactions suspected to be related to the medicinal product must be reported to BCPH by the treating physician (specified in the doctor’s declaration).	Any physician, pharmacist, dentist or midwife observing a serious or unexpected adverse reaction that could be due to the medicinal product with an ATU should notify the regional pharmacovigilance centre (CRPV) to which the reporter is geographically linked with or the addressee indicated in the PTU, which in turn conveys the information to ANSM.
	Who gathers pharmacovigilance data	A manufacturer, wholesaler or pharmacist who has been granted permission to supply an unregistered product is required to record all reported side effects (Art 3.17), which should be reported to the Inspectorate	The Belgian Centre for Pharmacovigilance for medicines for Human use (BCPH) is responsible for the coordination of various tasks related to pharmacovigilance. No further details (website page under construction)	As defined in the PTU, although mostly Regional Pharmacovigilance Centres (CRPV) collect and assess the information and transmit it to ANSM. The ATU may be suspended or withdrawn by the Director General of ANSM for public health reasons.

### Implementation of IV artesunate NPP in the Netherlands, Belgium and France

IV artesunate in the Netherlands has been introduced by a letter written by a healthcare professional internal medicines (P.J. de Vries, AMC) and an industrial pharmacist (C.K.W van Veldhuizen, ACE Pharmaceuticals) to medical professionals in the field of tropical medicines. The letter explained the status of the product and the procedures regarding how to order the product. In addition, the letter recommended that each hospital maintain an emergency stock of IV artesunate that is sufficient to treat at least one patient. In Belgium, prescribers at the Institute of Tropical Medicine in Antwerp initiated an NPP to gain access to Malacef by approaching ACE Pharmaceuticals, who in turn consulted the Federal Agency for Medicines and Health Products (FAMHP) for instalment of the programme in Belgium.

In France, temporary authorisation was initiated by ACE Pharmaceuticals in collaboration with the National Reference Centre for Malaria (CNR Paludisme). Authorisation was granted by ANSM after artesunate attained prequalification status from the WHO. Furthermore, a protocol for therapeutic use and data collection (PTU) was set up for IV artesunate by ANSM in collaboration with the distributor. This PTU serves three purposes: *i*) to provide physicians and pharmacists with information regarding the product and the conditions for its use, *ii*) to describe and organise pharmacovigilance, and *iii*) to organise patient follow-up and data collection and analysis.

### Availability of Malacef in the Netherlands, Belgium and France

**Figure 1:**
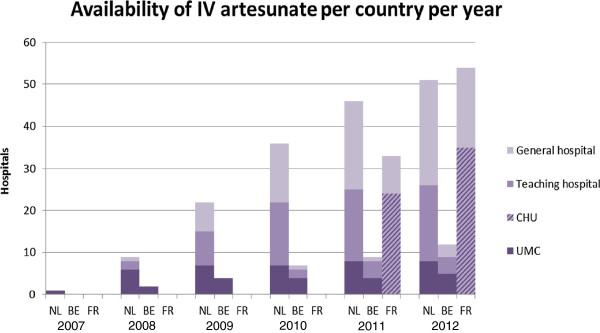
**Distribution of IV artesunate availability in the Netherlands (NE), Belgium (BE) and France (FR) by type of hospital.** CHU, centre hospitalier universitaire; UMC, university medical centre.

The availability of IV artesunate (Malacef) by country is shown in Figure 1. In the Netherlands, IV artesunate was initially almost exclusively stocked in UMCs, and this was quickly followed by teaching hospitals and general hospitals, covering a large area of the Netherlands. In Belgium, treatment using IV artesunate has been largely limited to specialised centres (42% UMCs, 33% teaching hospitals), whereas in France, 60% of the hospitals ordering IV artesunate were CHUs (17 out of 32 CHU in France).

**Figure 2:**
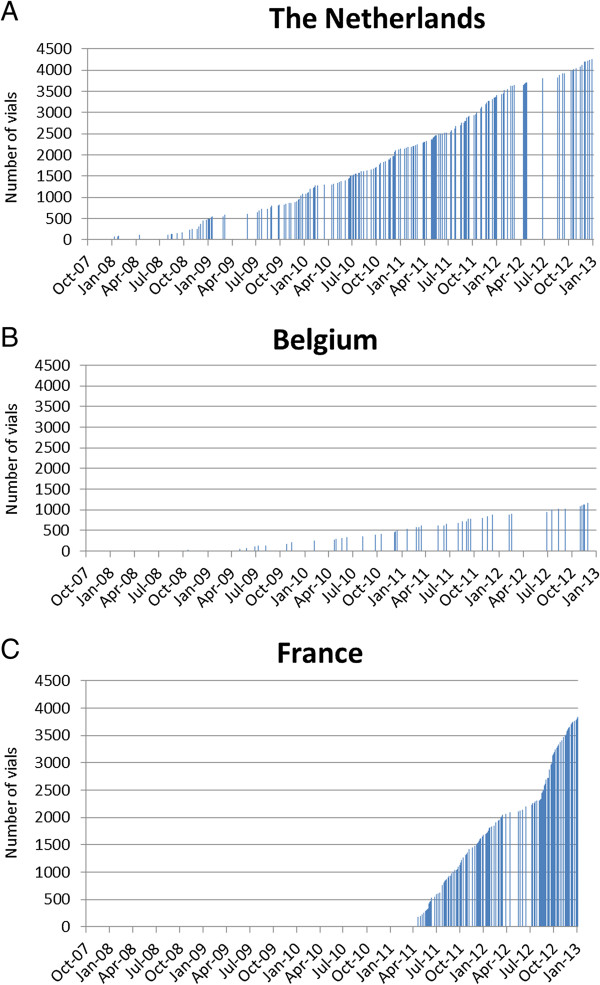
Cumulative number of vials of IV artesunate distributed to A) the Netherlands, B) Belgium and C) France based on distribution records from ACE Pharmaceuticals.

**Figure 3:**
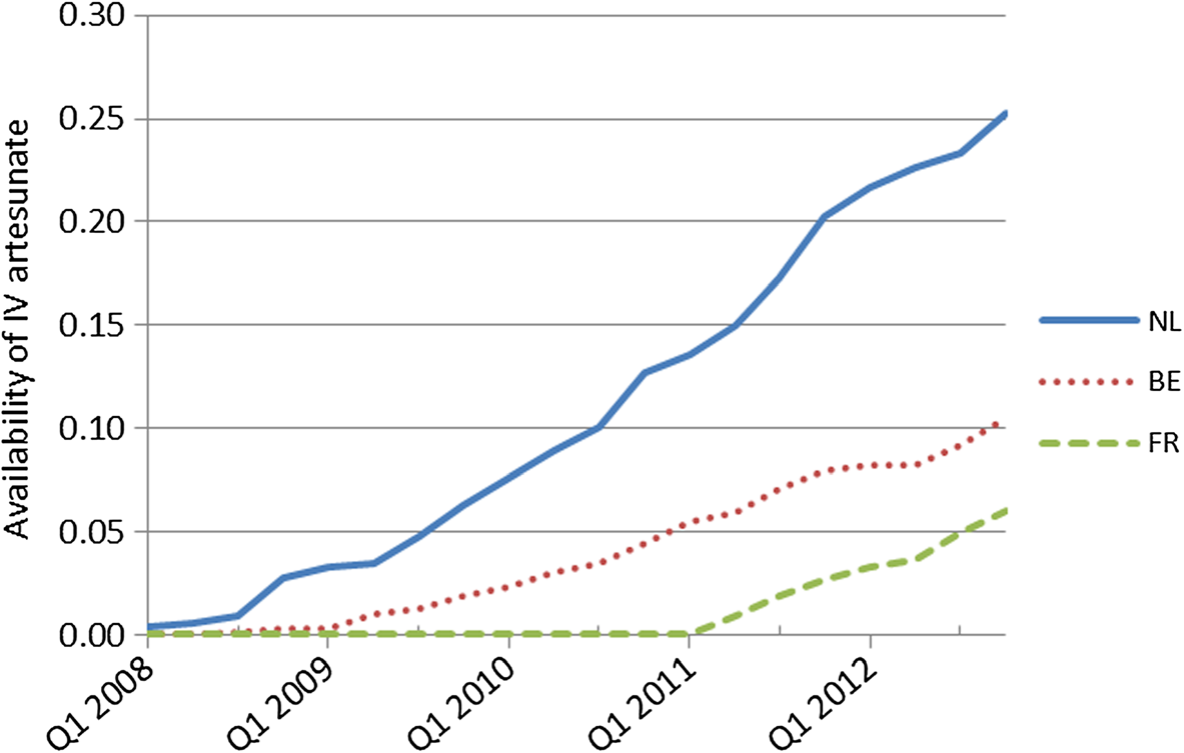
**Availability of IV artesunate per capita in the Netherlands (NL), Belgium (BE) and France (FR).** Availability was calculated using the following formula: [([the number of IV artesunate vials ordered per country)/ (the total population of that country)x1000]. Q1, first quarter of year.

The cumulative availability of IV artesunate vials for each country is plotted in Figure 2, and the availability per capita is plotted in Figure 3. In the Netherlands, the hospital stocks of IV artesunate per capita increased at a faster rate than in Belgium and France (Figure 3). At the end of 2012, availability per capita was 0.25 vials in the Netherlands, 0.1 vials in Belgium and 0.06 vials in France.

### Pharmacovigilance

In both Belgium and the Netherlands, pharmacovigilance in NPPs relies on the obligatory reporting of passively detected adverse events by individual physicians. Each prescription of a non-registered medicinal product must be accompanied by a statement from the physician who accepts responsibility for monitoring and reporting the drug’s efficacy and adverse events (if any occur). This passive notification system is quite different from the pro-active system in France, in which each newly treated patient must be authorised and must be actively followed with respect to pharmacovigilance.

Although this was not required, patients who were treated with IV artesunate in the Netherlands and Belgium from November 2007 through December 2010 were proactively traced in order to assess outcome [7]. Of the 68 patients who were treated (including 55 patients with severe malaria), two patients died (2/55 = 3.6%). The treatment was generally well-tolerated, and most of the recorded complications were compatible with the clinical findings associated with severe malaria and were present before IV artesunate treatment had started; therefore, these complications were not recorded as being related to the use of IV artesunate. It is noteworthy that seven patients developed an atypical form of haemolytic anaemia [7]. In the period of 2010 through 2012, only passive surveillance (i.e. spontaneous reporting) was performed, and no adverse events were reported. The haemolytic anaemia was initially detected by alert clinicians who were familiar with malaria and its treatment; the data collection was not designed specifically to capture this effect.

In France, the use of IV artesunate is subject to a protocol describing its therapeutic use, and pharmacovigilance is coordinated by the CNR Paludisme, Paris [14]. A total of 113 patients were treated for severe malaria with IV artesunate in the period from May 2011 through November 2012 [15]. The clinical course of the disease was favourable in 107 patients. The other six patients died in a very late stage of the disease while receiving IV artesunate therapy; two of these six patients were initially treated with IV quinine.

Twenty-seven patients experienced an episode of anaemia, including 15 cases of delayed and/or persistent haemolytic anaemia. One patient suffered from severe disseminated intravascular coagulation (DIC) with necrosis and amputation of fingers and legs in combination with a minimally conscious state. Furthermore, cytolytic hepatitis (three patients), cardiac involvement (transient bradycardia and QTc prolongation), rash (one patient), vision impairment (one patient), transient hyperkalaemia (one patient) and cerebellar ataxia (one patient) were also reported.

## Discussion

This article describes the roll out of Malacef (IV artesunate) under named patient programmes in three EU member countries. France has a system that uses proactive authorisation and surveillance, whereas Belgium and the Netherlands depend on the passive detection of adverse events. Most patients are treated at academic medical centres, but more specialised general hospitals also have IV artesunate in stock. The geographical distribution of Malacef throughout the Netherlands was relatively rapid, which is not surprising for a small country in which the professional groups are highly organised. In Belgium and France, the geographical distribution of Malacef proceeded more slowly. In Belgium, this is due to the fact that the use of Malacef is restricted to select specialised centres; in France, the slow distribution was likely due to a centralised procedure that is tightly controlled by the French government and requires strict and therefore lengthy application procedures.

The major limitations of NPPs include their non-centralised design and uncontrolled setting, particularly in the Netherlands and Belgium. However, our study shows that the investigator-initiated pharmacovigilance studies that were added to the NPPs in the Netherlands and Belgium [7] as well as in Germany [16] combined clinical expertise with a certain degree of serendipity sensitive enough to detect a new adverse event. Haemolysis is not uncommon in the reconvalescent phase of malaria [17]. Because the treating physicians see only individual cases, this form of haemolysis became apparent only after data pooling and trend analysis. In this particular case, the routine individual adverse event reporting in the Dutch and Belgian NPP legislations would have been too passive to identify this unusual adverse event.

On the other hand, formatted data collection studies such as clinical trials may only detect what they are designed to detect [18]. It is noteworthy that this adverse event was not described in the two largest trials that were conducted regarding both adults and children with severe malaria in endemic regions [2,3], although these trials were not designed to detect this late-onset event. It should be emphasised that the present study only reflects the situation in three EU member countries and that a named patient programme is not necessarily a suitable substitute for prospective clinical trials.

Collaborations between clinical researchers from academic institutions and pharmaceutical companies are often criticised due to concerns regarding financial ties to industry that may influence professional judgement with respect to patient care, medical research and/or education. However, academia-industry collaborations have also led to important and beneficial advantages in healthcare and are essential to the continued development of orphan drugs. Indeed, without this collaboration, IV artesunate might still not be available in Europe. Dutch clinicians were highly motivated to introduce IV artesunate in Europe because they witnessed first-hand the efficacy of IV artesunate in treating severe malaria. These clinicians formed a collaboration with a pharmaceutical company that specialises in the import of medical need products, and their efforts led to the legal import of IV artesunate from Guilin (in China) into the Netherlands and its export, after release, to other EU member countries.

NPPs have various positions within the European pharmaceutical legal and organisational frameworks [19]. The differences in the NPPs in the various EU member countries have led to differences in the availability of IV artesunate for treating European patients. The Netherlands has enjoyed a privileged position compared to the rest of Europe. Dutch patients have had access to Malacef since 2007, and Belgium followed in 2009; France received permission to use IV artesunate in April 2011. In the second half of 2012, Switzerland set up an NPP that was permitted by SwissMedic. Other countries are still debating this topic. This difference among EU member countries is based on the differences in national legislation and the approaches used to implement NPPs, as well as on various countries’ hesitation to use the product. For example, whether data collected from endemic regions can be extrapolated to the European population has been a subject of debate, and a comparative trial of parenteral artesunate versus quinine in the European patient is believed to be both unethical and unfeasible due to the relatively low incidence of malaria in Europe [4]. Nevertheless, the European Medicines Agency (EMA) and other national drug regulators depend on comparative trials as the primary instrument with which to collect the evidence needed for market authorisation, and NPPs are not embraced to the same extent as research studies. The French legislation even explicitly mentions that the use of medicinal products that are subject to an ATU cannot replace a clinical trial and that the goal of an ATU is not one of investigation. However, as revealed in this study, the pharmacovigilance component of NPPs can provide enormously valuable data, and their usefulness from a regulatory point of view should not be dismissed. For example, the safety data regarding one out of every seven European-approved orphan drugs were derive from such programmes [20]. NPPs represent a bridge between the controlled trial environment and real-world use and can even increase sensitivity for detecting adverse events.

Because the enrolment of patients in NPPs is usually initiated by physicians, the pharmaceutical companies usually have little influence on the process [6]. However, the manufacturer can play an important role in the collecting and sharing of data regarding the drug’s safety and efficacy. Although Guilin’s artesunate received WHO prequalification in 2010, it is worth noting that sterility issues of one batch of the product led to a worldwide “rapid alert” action on the initiative of the Dutch Pharmaceutical Inspection in May 2012 which amongst others resulted in a recall in several countries. Therefore, before being released for distribution, each batch of imported IV artesunate is subjected to a series of both physicochemical quality controls and sterility tests in accordance with European Pharmacopeia requirements. Furthermore, drug distribution must be controlled, and supplies must be transparent to ensure that there is no trading. Finally, pharmacovigilance data must be collected, and risks should be anticipated as much as possible [6].

In France, although it is both time-consuming and tedious, a proactive authorisation system is in place, and pharmacovigilance is designed to be much stricter with respect to data collection. In Belgium and the Netherlands, on the other hand, the NPPs were implemented relatively quickly, and surveillance has relied on reporting by physicians. Without guidance or legal enforcement to actively require physicians to report adverse event, this system is prone to underreporting [21]. In addition, without aggregating individual observations and/or the sharing of knowledge, identifying a potential link between an apparent adverse reaction and a drug is more difficult. Indeed, if an active follow-up had not been initiated in Belgium and the Netherlands, the haemolytic anaemia that was recently described in seven patients would not have been detected. Although these reported cases of late-onset haemolysis recovered without sequelae, and although anaemia is likely attributable to the malaria rather than to the use of IV artesunate [22], additional safety data are needed.

The present study illustrates that similar to full-market authorization, NPPs require a reliable pharmacovigilance system that captures all data, follows all patients and allows scientists to study and identify adverse event patterns. Because of the rarity of the disease and patients, it is imperative that all patients who are treated in an NPP can be easily identified and monitored at all times, and patients should therefore be registered in order to minimize any loss of data. This can be achieved by requiring an electronic database to keep track of electronic prescriptions for each patient who receives treatment with an unauthorized medicine. The prescribing physician can also register his/her medical license number and can file the prescription with a code that will allow the physician to identify and track the patient. Using this approach, both the patient and the physician are traceable at all times. In addition, the unauthorised medicine would only be dispensed by a pharmacist when such an electronic prescription is available, and this should be strictly controlled (and the pharmacist should be sanctioned if the proper procedures are not followed). The manufacturer and/or distributor can play a pivotal role in facilitating the connection between the product’s distribution and its registration in the database.

New legislation should be introduced throughout the EU in order to both optimize and harmonize the pharmacovigilance of orphan and other unregistered products. Harmonization should ensure equal availability of the treatment and therapeutic benefit to all Europeans. Efforts must be made to both simplify and accelerate access to NPP drugs particularly in emergency situations such as severe malaria in order to save valuable time for patients in need. This ambitious yet attainable goal can only be achieved through close cooperation among prescribers, pharmacists, academia, industry and in particular the drug regulators, who should take the lead. In this respect, we feel that the EMA has two important roles to play: first, in facilitating and implementing these new EU rules, and second, in exploring how NPPs can contribute to the registration of orphan drugs.

## Conclusions

IV artesunate has rapidly gained its place in the treatment of severe malaria in the Netherlands, and other countries are following (albeit hesitantly). This hesitation is due to both differences in the implementation of NPP legislation and the knowledge gap with respect to using IV artesunate to treat European patients.

## Abbreviations

ANSM: Agence National de Securité du Medicament et des Produits de Santé; ATU: Temporary Authorisation for Use; CBG-MEB: Medicines Evaluation Board; CHU: Centre Hospitalier Universitaire; CNR Paludisme: National Reference Centre for Malaria; CUP: compassionate use programme; EMA: European Medicines Agency; FAMHP: Federal Agency for Medicines and Health Products; GMP: Good Manufacturing Practices; IV: Intravenous; NPP: named patient programme; PTU: protocol for therapeutic use; UMC: University Medical Centre, WHO, World Health Organization.

## Competing interests

ARKV is a PhD fellow employed by ACE Pharmaceuticals; CKWvV is pharmacist employed by ACE Pharmaceuticals, and PJdV is medical and scientific advisor to ACE Pharmaceuticals.

## Authors’ contributions

ARKV and PJdV conceived and designed the study and analysed and interpreted the data. CKWvV acquired the data and was involved in drafting the manuscript. All authors were involved in writing and revising the manuscript.
